# Evaluation of changes in shoulder balance and prediction of final shoulder imbalance during growing-rod treatment for early-onset scoliosis

**DOI:** 10.1186/s12891-021-04221-9

**Published:** 2021-04-14

**Authors:** Ziyang Liu, Tie Liu, Yong Hai, Lingyun Wu, Junrui Jonathan Hai, Kang Gao, Xuanrong Guo, Honghao Yang, Nan Kang, Fan Zhao

**Affiliations:** 1 Department of Orthopedic Surgery, Beijing Chaoyang Hospital, Capital Medical University, Gongti North Rd, No. 8, Beijing, 100020 China; 2 Karolinska Institutet, Stockholm, Sweden; 3 The High School Affiliated to Renmin University of China, Beijing, China; 4 Capital Medical University, Beijing, China; 5 University of Wisconsin Madison, Madison, WI USA

**Keywords:** Shoulder balance, Early-onset scoliosis, Growing rod, Spinal fusion, Postoperative shoulder imbalance

## Abstract

**Background:**

Obtaining and maintaining final shoulder balance after the entire treatment course is essential for early-onset scoliosis (EOS) patients. The relatively small number of growing-rod (GR) graduates who complete final fusion has resulted in an overall paucity of research on the GR treatment of EOS and a lack of research on the shoulder balance of EOS patients during GR treatment.

**Methods:**

Twenty-four consecutive patients who underwent GR treatment until final fusion were included. Radiographic shoulder balance parameters, including the radiographic shoulder height (RSH), clavicle angle (CA), and T1 tilt angle (T1T), before and after each step of the entire treatment were measured. Shoulder balance changes from GR implantation to the last follow-up after final fusion were depicted and analysed. Demographic data, surgical-related factors, and radiographic parameters were analysed to identify risk factors for final shoulder imbalance. The shoulder balance of patients at different time points was further analysed to explore the potential effect of the series of GR treatment steps on shoulder balance.

**Results:**

The RSH showed substantial improvement after GR implantation (*P* = 0.036), during the follow-up period after final fusion (*P* = 0.021) and throughout the entire treatment (*P* = 0.011). The trend of change in the CA was similar to that of the RSH, and the T1T improved immediately after GR implantation (*P* = 0.037). Further analysis indicated that patients with shoulder imbalance before final fusion showed significantly improved shoulder balance after fusion (*P* = 0.045), and their RSH values at early postfusion and the final follow-up did not show statistically significant differences from those in the prefusion shoulder balance group (*P* > 0.05). Early postfusion shoulder imbalance (odds ratio (OR): 19.500; 95% confidence interval (CI) = 1.777–213.949; *P* = 0.015) was identified as an independent risk factor for final shoulder imbalance.

**Conclusions:**

Shoulder balance could be improved by GR implantation but often changes during the multistep lengthening process, and the final result is relatively unpredictable. Final fusion could further adjust the prefusion shoulder imbalance. Focusing on the prefusion shoulder balance of GR graduates and providing patients with early shoulder balance after fusion might be necessary.

**Supplementary Information:**

The online version contains supplementary material available at 10.1186/s12891-021-04221-9.

## Background

Good surgical outcomes in patients with scoliosis include the sufficient correction of curves in three planes and prevention of further progression, as well as good shoulder balance and trunk balance [1–3]. Shoulder balance influences not only patient appearance and satisfaction but also potential complications, such as adding-on, trunk shifting, and reoperation [2–7]. Thus, preventing postoperative shoulder imbalance requires particular attention. Previous studies have reported various risk factors for postoperative shoulder imbalance but are mostly based on adolescent idiopathic scoliosis [2, 4, 5, 8–11]. The risk factors for postoperative shoulder imbalance in early-onset scoliosis (EOS) are still unknown.

Growing-rod (GR) surgery is indicated for EOS patients when curves are of sufficient magnitude and when conservative treatment methods have failed [12–14]. The entire GR treatment has been proven to obtain optimal correction results and excellent growth potential [12–16], but the effect on shoulder balance has remained understudied. Uzümcügil et al. [17] and Atici et al. [18, 19] evaluated the effect of GR on shoulder balance during treatment periods. However, only a few patients in previous studies were GR graduates who underwent final fusion. Final fusion is considered to be the first choice for the last step of GR treatment for most EOS patients, and although it remains controversial, most patients who have completed final fusion have achieved definitive curative effects [20–22]. Focusing on GR graduates who have completed final fusion is of great significance for investigating the effect of GR on final shoulder balance. In this study, all patients included were GR graduates who had completed final fusion, a distinction lacking in previous studies.

The purpose of this study was to explore the effect of GR on shoulder balance in GR graduates and to identify possible predictors for final shoulder balance.

## Methods

### Inclusion criteria

Twenty-four consecutive EOS patients who underwent the entire series of surgical treatments, including GR implantation, multiple lengthening surgeries, and final fusion, at our institution from March 2008 to July 2018 were studied. Patients who underwent other spinal surgeries were excluded.

### Parameter measurements

We obtained radiographic parameters by measuring the standing full-length spine films taken at predetermined time points: before and early after GR implantation, before and after multiple lengthening surgeries, before and early after final fusion, and at the last follow-up. Two observers performed measurements independently using the Picture Archiving and Communication System (PACS) technology (GE Medical Systems, 2006).

The radiographic shoulder height (RSH) was obtained by measuring the height difference of the soft tissue shadow above the acromioclavicular joint, and absolute RSH values were used to represent the severity of shoulder imbalance [23, 24]. Shoulder balance conditions were categorised based on the RSH: balance (< 2 cm), imbalance (mild imbalance (2–3 cm), and severe imbalance (> 3 cm)) [2, 4, 5, 9, 10]. In addition, the T1 tilt angle (T1T) was defined as the angle between the upper endplate of the T1 vertebral body and the horizontal line, while the clavicle angle (CA) was defined as the angle between the tangent line touching the highest point of each clavicle and the horizontal line [3–6, 8–10, 23].

The major curve (MC), the proximal thoracic curve (PTC), the cervical 7 plumb line-centre sacral vertical line (C7PL-CSVL), the cervical 7 plumb line-sagittal vertebral axis (C7PL-SVA), thoracic kyphosis (TK), and lumbar lordosis (LL) were also measured.

### Satisfaction questionnaire

The questionnaire (see supplementary information) was modified from the Spinal Appearance Questionnaire (SAQ) [25] and that of Kuklo et al. [23] according to the characteristics of GR treatment to assess parental perception and satisfaction of patients’ shoulder balance. It was distributed to fifteen parents of the included patients who had finished the treatment in the last 6 years.

### Statistical analysis

Intraclass correlation coefficients (ICCs) were calculated to assess the interobserver and intraobserver reliability of the measurements performed by the observers. Categorical variables were compared by Fisher’s exact test. Comparisons between two different groups were performed by the Mann-Whitney U test or independent t-test; comparisons between different time points were performed by repeated-measures ANOVA. Multiple time point analyses were performed to describe the changes in shoulder balance during GR treatment.

To investigate the relationships between patient characteristics and clinical outcomes, variables that exhibited significant differences in univariate comparisons (the threshold for variables included in the multivariate regression analysis was *P* = 0.10) were included in the stepwise logistic regression to identify the risk factors for shoulder imbalance at follow-up.

Analyses were performed by SPSS 25.0 (SPSS, Chicago, IL). A *P* value of less than 0.05 was considered statistically significant.

## Results

### Demographics

Demographic data are summarised in Table 1. Due to failure to control spinal deformity, severe coronal decompensation and rod breakage, single rod in six patients were replaced with dual rods during the lengthening process. Eleven patients had dual rods before final fusion, and single-rod configurations remained in thirteen patients.

**Table 1:** Demographics

Descriptive items	Mean ± SD or Number (%)
Sex	
Male	6 (25.0%)
Female	18 (75.0%)
Diagnosis	
Idiopathic	10 (41.7%)
Congenital	9 (37.5%)
Other	5 (20.8%)
Age (implantation) (year)	9.8 ± 2.3
Age (fusion) (year)	13.0 ± 1.5
Risser sign (implantation) (0/1)	
0	21 (87.5%)
1	3 (12.5%)
Risser sign (fusion) (4/5)	
4	4 (16.7%)
5	20 (83.3%)
Treatment duration (year)	3.2 ± 1.4
No. of lengthening surgeries	2.5 ± 1.5
Implantation rod(s)	
Single	19 (79.2%)
Dual	5 (20.8%)
UIV (Growing rods)	
T2 and above	16 (66.7%)
T3 and below	8 (33.3%)
UIV (Fusion)	
T2 and above	14 (58.3%)
T3 and below	10 (41.7%)
LIV (Growing rods)	
L3 and above	10 (41.7%)
L4 and below	14 (58.3%)
LIV (Fusion)	
L3 and above	17 (70.8%)
L4 and below	7 (29.2%)
Upper instrument (Growing rods)	
Hook/hybrid	16 (66.7%)
No hook	8 (33.3%)
Upper instrument (Fusion)	
Hook/hybrid	5 (20.8%)
No hook	19 (79.2%)

*UIV* upper instrumented vertebrae; *LIV* lower instrumented vertebrae

### Radiographic measurements

Radiographic measurements are summarised in Table 2. The analysis revealed significant improvement in the MC throughout the entire treatment (*P* < 0.001). In addition, the average PTC and TK significantly decreased (PTC: *P* < 0.001; TK: *P* = 0.041).

**Table 2:** Radiographic parameters from preimplantation to final follow-up for the entire study population

	MC (°)	PTC (°)	TK (°)	LL (°)	C7PL-CSVL (cm)	C7PL-SVA (cm)
Preimplantation	96 ± 22	43 ± 18	47 ± 24	48 ± 14	3.0 ± 2.6	3.0 ± 2.0
Postimplantation	51 ± 20	30 ± 13	33 ± 15	41 ± 14	2.4 ± 1.8	3.2 ± 1.8
Prefusion	59 ± 20	30 ± 12	41 ± 15	57 ± 14	2.4 ± 1.7	2.7 ± 2.1
Follow-up	38 ± 17	21 ± 8	35 ± 12	52 ± 12	2.7 ± 1.6	3.2 ± 2.3

*MC* major curve; *PTC* proximal thoracic curve; *TK* thoracic kyphosis; *LL* lumbar lordosis; *C7PL* cervical 7 plumb line; *CSVL* centre sacral vertical line; *SVA* sagittal vertebral axis

### Changes in shoulder balance

The intraobserver ICC was 0.88, and the interobserver ICC was 0.83 [26].

**Fig. 1:**
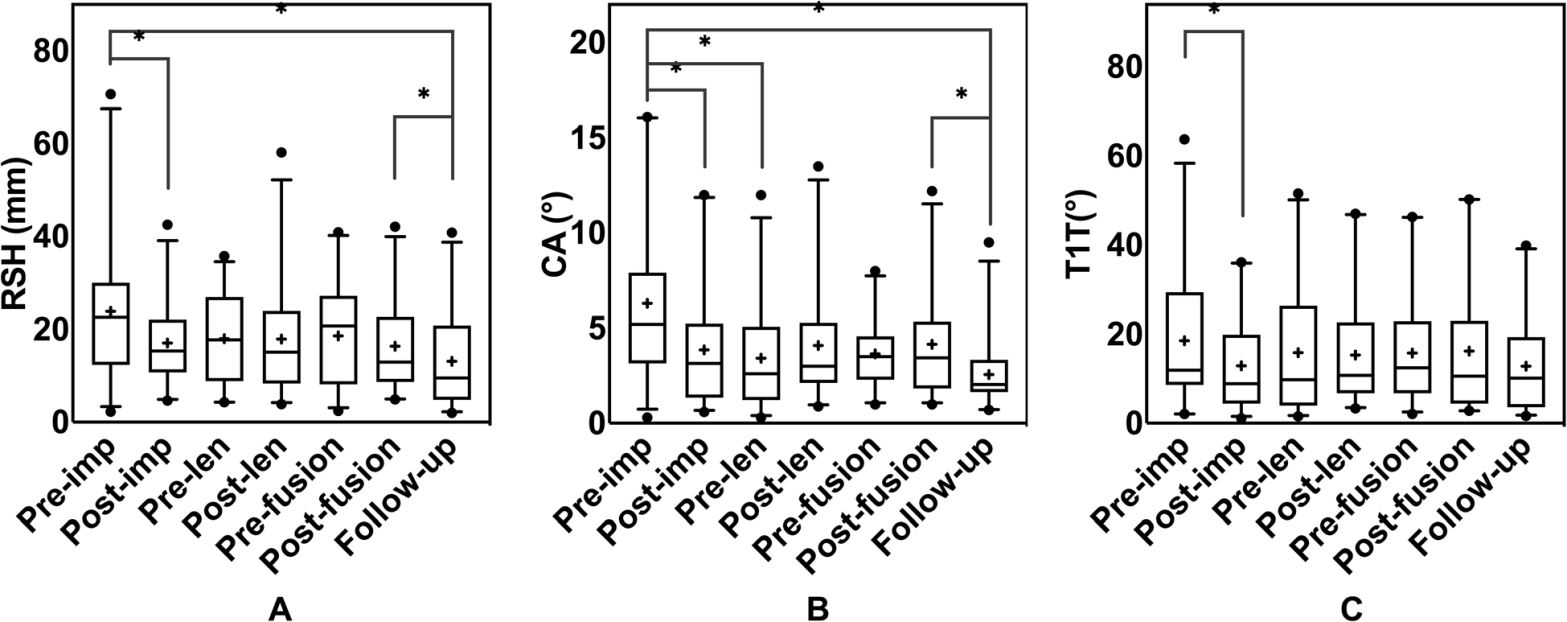
Changes in the radiographic shoulder balance of EOS patients throughout the entire growing rod treatment period. **a** RSH (**b**) CA (**c**) T1T.*Statistically significant. +: *mean value*. ●: *extreme values*

RSH values at different time points are plotted in Fig. 1a. The average RSH at preimplantation, early postimplantation, prefusion, early postfusion, and final follow-up was 23.8 ± 16.0 mm, 17.1 ± 8.6 mm, 18.6 ± 11.0 mm, 16.4 ± 9.5 mm, and 13.2 ± 10.7 mm, respectively. The analysis revealed statistically significant improvements in the RSH after GR implantation (*P* = 0.036), during the follow-up period after final fusion (*P* = 0.021), and throughout the entire treatment (*P* = 0.011). The RSH during the lengthening procedures, however, did not change significantly (*P* > 0.05), and there was no significant difference in the RSH between before fusion and before implantation (*P* > 0.05). The changes in the CA and T1T are shown in Fig. 1b, c. As shown in Fig. 1b, the average CA decreased significantly after GR implantation (*P* = 0.008), during the follow-up period after fusion (*P* = 0.024), and after the entire treatment (*P* = 0.003). The T1T showed a significant decrease after GR implantation (*P* = 0.037; Fig. 1c).

**Fig. 2:**
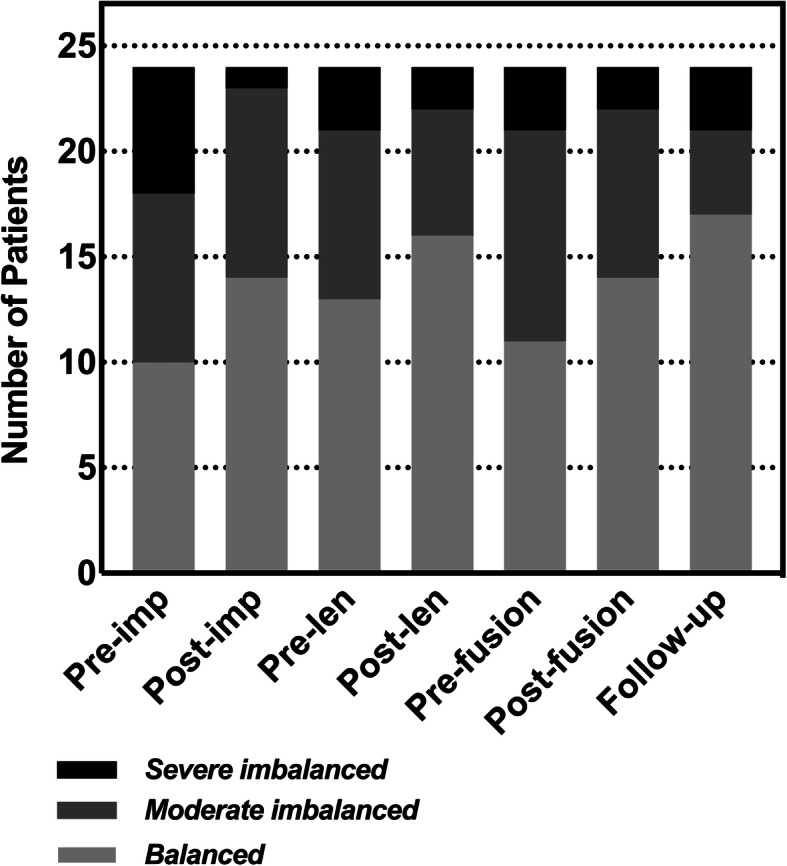
Number of patients with different shoulder balance statuses during the growing rod treatment period

Figure 2 shows a plot of the number of patients with balanced shoulders at different time points. At the five time points mentioned above, the number of patients with balanced shoulders was 10 (42%), 14 (58%), 11 (46%), 14 (58%), and 17 (71%).

**Fig. 3:**
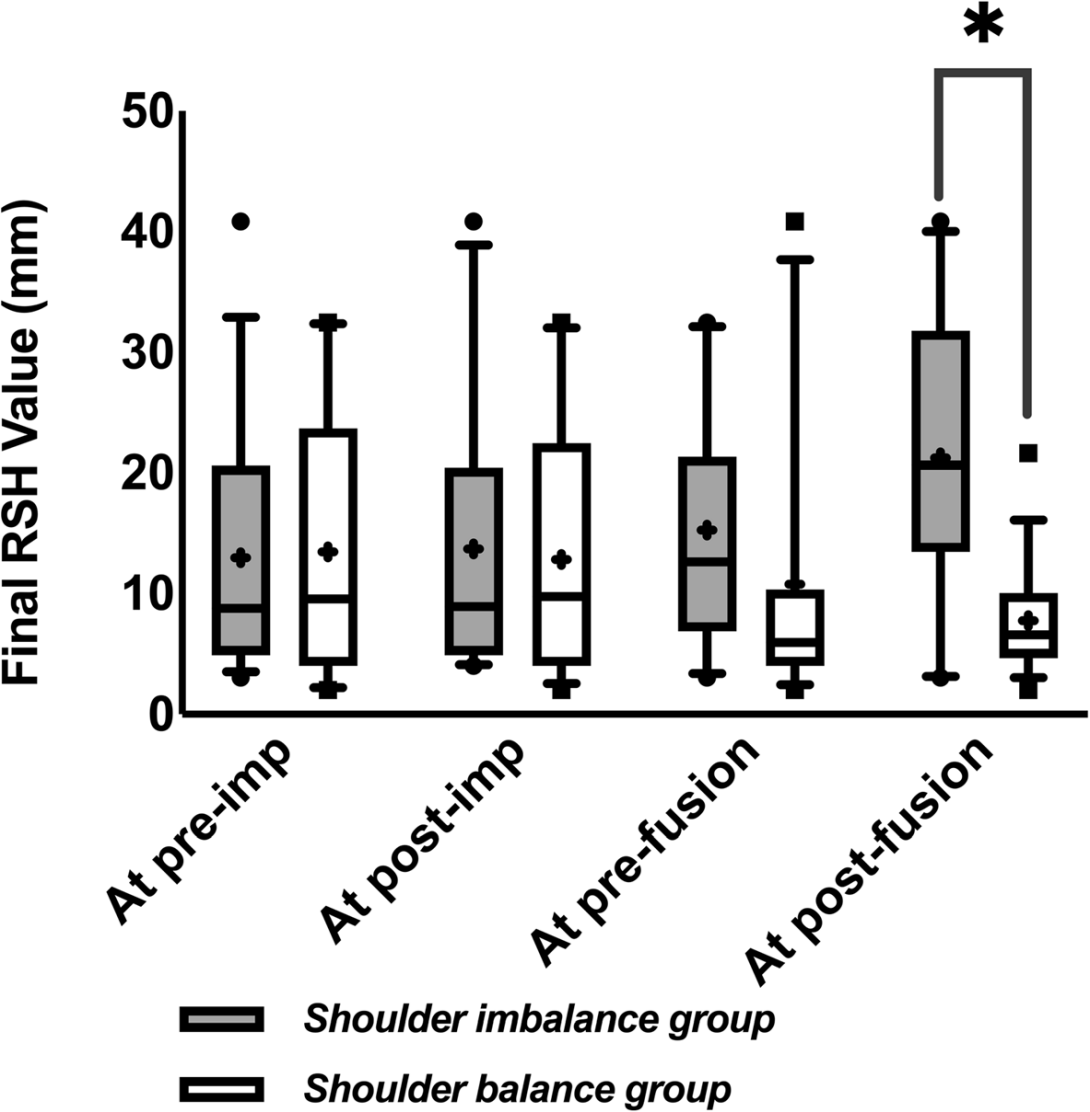
Comparison of the final RSH between patients grouped by shoulder balance status at preimplantation, postimplantation, prefusion, and early postfusion. Grey bar: shoulder imbalance group. White bar: shoulder balance group. *Statistically significant. +: *mean value*. ●■: *extreme values*

**Fig. 4:**
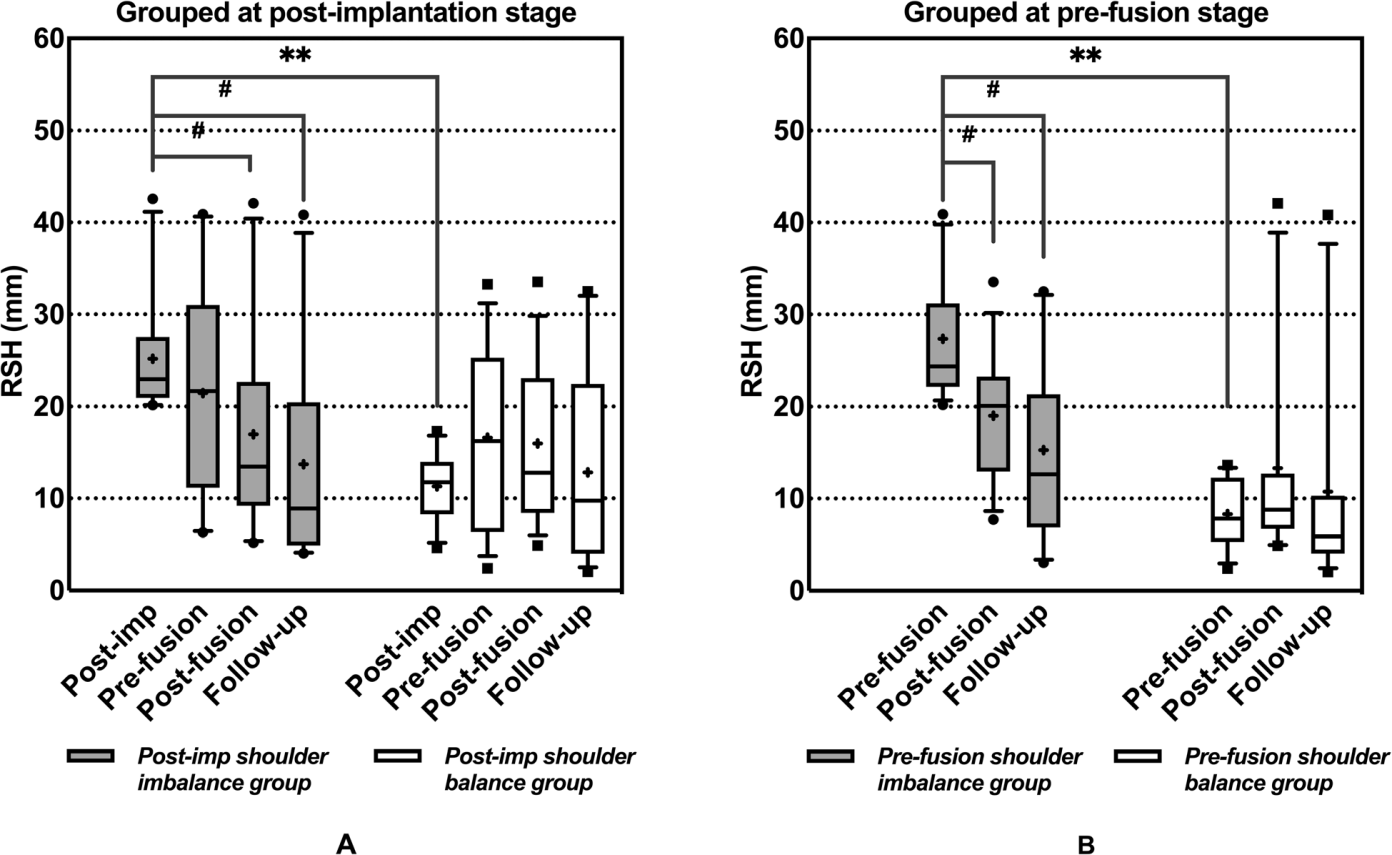
Changes in the RSH of patients in the shoulder balance group and shoulder imbalance group. Patients grouped at (**a**) postimplantation and (**b**) prefusion. Grey bar: shoulder imbalance group. White bar: shoulder balance group. *Statistically significant differences between groups (**P < 0.001). #Statistically significant improvement in the group. +: mean value. ●■: extreme values

To further identify the time points with potentially predictable final shoulder balance and further analyse the potential impact of each step of GR treatment on shoulder balance, the patients were divided into two (shoulder balance and shoulder imbalance) groups at the aforementioned five time points. Figure 3 shows a significant difference in the final RSH between the early postfusion shoulder balance and imbalance groups (*P* = 0.008). Further analysis results of the postimplantation groups are shown in Fig. 4a. Although the RSH in the postimplantation shoulder imbalance group did not show a significant improvement from postimplantation to prefusion (*P* > 0.05), the RSH in the two groups no longer showed a significant difference between prefusion and the final follow-up (P > 0.05). The results of further analysis of the prefusion groups are shown in Fig. 4b. For patients with shoulder imbalance before fusion, their RSH was improved immediately after fusion (*P* = 0.045), and there was no longer a significant difference from the prefusion shoulder balance group (*P* > 0.05).

### Variable analysis

Seven (29%) patients developed radiographic shoulder imbalance at the last follow-up based on the diagnostic criteria. The results of the univariate analysis showed that potential risk factors for shoulder imbalance included late acceptance of GR implantation (*P* = 0.094) and final fusion (*P* = 0.091), upper instrumented vertebrate (UIV) below T2 at final fusion (*P* = 0.085), prefusion sagittal imbalance (*P* = 0.074), a large early postfusion RSH (*P* = 0.002), a large early postfusion CA (*P* = 0.082), and a rating of radiographic shoulder imbalance (*P* = 0.009) (Table 3). In the multivariate logistic stepwise regression analysis, early postfusion shoulder imbalance (odds ratio (OR): 19.500; 95% confidence interval (CI) = 1.777–213.949; *P* = 0.015) was found to be an independent risk factor for final shoulder imbalance.

**Table 3:** Univariate analysis of shoulder imbalance risk factors

Parameters	Balanced shoulder	Imbalanced shoulder	***P***
Demographics			
Sex (male/female)	4/13	2/5	1.000
Diagnosis (congenital/not)	5/12	4/3	0.356
Age at implantation	9.3 ± 2.3	11.0 ± 1.6	**0.094**
Age at fusion	12.7 ± 1.5	13.9 ± 1.2	**0.091**
Risser sign at implantation (0/1)	15/2	6/1	1.000
Risser sign at fusion (4/5)	3/14	1/6	1.000
Treatment duration (y)	3.4 ± 1.6	2.8 ± 1.0	0.429
Left/right curve	4/13	3/4	0.374
Surgery-related			
No. of lengthening surgeries	2.5 ± 1.7	2.4 ± 1.0	0.885
Single/dual rod(s)			
Implantation	13/4	6/1	1.000
Prefusion	8/9	5/2	0.386
UIV(T2 and above/T3 and below)			
Growing rods	12/5	4/3	0.647
Fusion	12/5	2/5	**0.085**
LIV(L3 and above/L4 and below)			
Growing rods	8/9	2/5	0.653
Fusion	12/5	5/2	1.000
Upper instrument (hook or hybrid/no hook)			
Growing rods	11/6	5/2	1.000
Fusion	3/14	2/5	0.608
Fixation of the apical region/not at fusion	11/6	3/4	0.393
Osteotomy/not at fusion	3/14	2/5	0.608
Radiographic			
MC Cobb angle (°)			
Preimplantation	98 ± 20	93 ± 26	0.637
Postimplantation	53 ± 18	45 ± 26	0.359
Prefusion	60 ± 19	57 ± 24	0.756
Follow-up	41 ± 17	32 ± 18	0.252
Change	57 ± 12	61 ± 17	0.506
PTC Cobb angle (°)			
Preimplantation	44 ± 20	42 ± 15	0.815
Postimplantation	30 ± 14	29 ± 10	0.947
Prefusion	30 ± 14	30 ± 9	0.890
Follow-up	21 ± 8	21 ± 6	0.895
Change	22 ± 14	21 ± 13	0.887
Flexibility (preimplantation)			
PTC	38 ± 27	29 ± 12	0.433
MC	19 ± 17	19 ± 15	0.976
Thoracic kyphosis			
Preimplantation	46 ± 25	52 ± 25	0.575
Postimplantation	34 ± 15	31 ± 17	0.689
Prefusion	39 ± 15	47 ± 12	0.239
Follow-up	33 ± 12	39 ± 12	0.272
Change	13 ± 22	13 ± 19	0.983
Lumbar lordosis			
Preimplantation	48 ± 15	48 ± 14	0.980
Postimplantation	40 ± 13	44 ± 16	0.511
Prefusion	56 ± 14	61 ± 13	0.384
Follow-up	53 ± 10	52 ± 16	0.888
Change	−5 ± 18	−4 ± 17	0.955
C7PL-CSVL (cm)			
Preimplantation	3.1 ± 2.4	3.0 ± 3.2	0.936
Postimplantation	2.4 ± 1.6	2.5 ± 2.4	0.896
Prefusion	2.5 ± 1.6	2.1 ± 1.8	0.559
Follow-up	2.9 ± 1.6	2.2 ± 1.7	0.392
Change	0.2 ± 3.4	0.8 ± 3.0	0.699
C7PL-SVA (cm)			
Preimplantation	2.7 ± 2.0	3.7 ± 2.0	0.303
Postimplantation	3.3 ± 1.9	2.9 ± 1.4	0.641
Prefusion	2.2 ± 1.8	3.9 ± 2.5	**0.074**
Follow-up	3.0 ± 2.4	3.5 ± 2.0	0.685
Change	−0.3 ± 3.2	0.2 ± 2.6	0.722
Shoulder Balance			
RSH (mm)			
Preimplantation	23.7 ± 16.1	24.2 ± 16.9	0.942
Postimplantation	16.7 ± 5.9	18.1 ± 13.8	0.807
Prelengthening	18.6 ± 10.2	16.7 ± 9.1	0.680
Postlengthening	18.4 ± 13.6	16.9 ± 6.7	0.792
Prefusion	18.3 ± 11.8	19.5 ± 9.8	0.806
Postfusion	12.5 ± 6.4	25.8 ± 9.5	**0.002**
Follow-up	7.3 ± 4.0	27.5 ± 7.7	**< 0.001**
RSB classification (balanced/unbalanced)			
Preimplantation	7/10	4/3	0.659
Postimplantation	10/7	4/3	1.000
Prelengthening	9/8	5/2	0.653
Postlengthening	11/6	5/2	1.000
Prefusion	9/8	2/5	0.386
Postfusion	13/4	1/6	**0.009**
Follow-up	17/0	0/7	**< 0.001**
Other Parameters			
CA (°)			
Preimplantation	6.8 ± 4.2	5.1 ± 4.2	0.392
Postimplantation	4.0 ± 2.9	3.6 ± 4.2	0.814
Prefusion	3.8 ± 2.0	3.3 ± 1.3	0.926
Postfusion	3.4 ± 2.7	5.8 ± 3.3	**0.082**
Follow-up	1.9 ± 0.9	4.3 ± 2.6	**0.044**
T1T (°)			
Preimplantation	18.8 ± 15.9	17.9 ± 15.1	0.900
Postimplantation	12.7 ± 10.5	13.6 ± 11.8	0.846
Prefusion	16.1 ± 13.1	14.9 ± 9.5	0.822
Postfusion	15.0 ± 15.1	19.3 ± 13.5	0.523
Follow-up	11.3 ± 11.8	16.5 ± 7.2	0.295

*RSH* radiographic shoulder height; *RSB* radiographic shoulder balance; *UIV* upper instrumented vertebrae; *LIV* lower instrumented vertebrae; *C7PL* cervical 7 plumb line; *CSVL* centre sacral vertical line; *SVA* sagittal vertebral axis; *MC* major curve; *PTC*,proximal thoracic curve

Bold numbers indicate *P*<0.10

### Questionnaire

Thirteen (87%, 13/15) valid questionnaires were received. Four (31%) patients had radiographic shoulder imbalance at the last follow-up and were considered ‘mildly unbalanced’. Nine (69%) patients were corrected to radiographic shoulder balance (6 (67%) were considered ‘balanced’, and three (33%) were considered ‘mildly unbalanced’). There was no statistically significant difference in the parental evaluation of shoulder balance between patients with radiographic shoulder balance and imbalance (*P* = 0.228). Interestingly, all parents (100%) agreed that GR treatment improved the shoulder balance of the patients. All parents (100%) were satisfied with the final shoulder condition of patients (9 (69%) chose ‘basically satisfied’, 4 (31%) chose ‘very satisfied’): Parental satisfaction did not show statistically significant differences between patients with final radiographic shoulder imbalance and final radiographic shoulder balance (*P* = 0.109).

## Discussion

Immediately after GR implantation, the mean RSH showed a significant decrease (*P* = 0.036; Fig. 1a), and the number of patients with shoulder balance increased from 10 to 14. Furthermore, the CA (*P* = 0.008) and T1T (*P* = 0.037) improved immediately after GR implantation. These results suggested that GR implantation improved shoulder balance. Studies by Uzümcügil et al. [17] and Atici et al. [18, 19] reported that GR had a positive effect on shoulder balance. Our findings are consistent with these previous reports. Adjustment of the shoulder balance is part of adjacent compensation, which is often triggered by deformity correction [27, 28]. In view of that, we attributed the adjustment of shoulder balance to the significant correction of the PTC and MC (Table 2) [2, 10, 29].

After multiple lengthening procedures, there was no longer a statistically significant difference in the RSH between the patients with preimplantation shoulder balance and those with shoulder imbalance (Fig. 4a), and there was no statistically significant difference in the radiographic shoulder balance parameters of all patients between before fusion and before implantation (*P* > 0.05; Fig. 1abc). In general, regular lengthening not only prevents the deterioration of spinal deformities and treats complications but also unleashes spine growth potential and readjusts the balance [20, 30, 31]. However, according to the aforementioned studies and the current study, regular lengthening had no significant effect on improving shoulder balance.

Final fusion was recommended for most GR graduates, but in previous studies, only a small number of patients were GR graduates who underwent final fusion [17–19]. In this study, all patients included were GR graduates who completed final fusion and had relatively complete follow-up data. Further analysis showed that final fusion significantly improved shoulder balance in patients with prefusion shoulder imbalance (Fig. 4b). In addition, the number of patients with severe shoulder imbalance decreased after final fusion (Fig. 2). These results might suggest that final fusion enabled rebalancing in patients with shoulder imbalance. In general, final fusion can help patients not only stop the high complication rate during multiple lengthening procedures and further correct deformities but also adjust trunk imbalance and achieve better biomechanical fixation [20, 22, 30, 31]. Furthermore, it is encouraging that final fusion achieved good results for improving prefusion shoulder imbalance according to the results.

Our study revealed a significant difference in the RSH at the last follow-up compared with immediately after fusion (*P* = 0.021; Fig. 1a), and the number of patients with balanced shoulders increased during the follow-up period after fusion (Fig. 2). A similar trend was found in the CA (*P* = 0.024; Fig. 1b). These results might suggest that GR graduates might experience ‘spontaneous correction of shoulder balance’ after final fusion. Although there is no uniform language that describes this conjecture in the literature, this phenomenon has been reported in patients with adolescent idiopathic scoliosis undergoing spinal fusion [4, 10, 23, 27]. However, although it has not been previously reported in EOS patients, it might exist in EOS patients who complete final fusion given the similarities between segmental fusion for other types of scoliosis surgery and final fusion. The selective instrumented segments in final fusion often preserve motion segments for the postoperative spine, which enables postoperative balance restoration [6, 32–35]. In addition, according to previous reports, the early postoperative shoulder balance status might be affected by postoperative pain and malposture, which might improve once postoperative pain is relieved [35].

Perfect postoperative neutral shoulder balance is not always achieved, but this does not mean that shoulder balance is not favourably improved by surgery. A certain degree of neutral shoulder bias can exist even in the normal population. Previous studies on shoulder balance parameters in normal people without scoliosis have shown that most people’s shoulder balance is not in the absolute neutral position, but the vast majority of people are at approximately the absolute neutral level [28, 36–38]. Currently, the maximum sample size that has been used in a study of a normal population (232 adolescents) has shown that the average RSH in the normal population is 0.9 cm [28]. In our study, the patients’ mean RSH improved from 23.8 ± 16.0 mm at preimplantation to 13.2 ± 10.7 mm at the final follow-up, showing a statistically significant improvement and tending toward the neutral shoulder balance level in the general population.

In this study, shoulder imbalance early after fusion was identified as a definite risk factor for final shoulder imbalance in GR graduates. Early postoperative RSH imbalance was previously reported to be a primary predictor of final shoulder imbalance [2]. Notably, early postoperative shoulder imbalance as an independent risk factor seems to contradict the spontaneous correction of shoulder balance after final fusion. However, this study demonstrated limited spontaneous recovery of shoulder balance after final fusion and that not every patient with early postoperative shoulder imbalance eventually showed an improvement, which is consistent with previous literature [4, 10, 27]. Other potential risk factors, including the UIV of final fusion, age at implantation, age at final fusion, and prefusion C7PL-SVA, were identified as not statistically significant. Whether the above factors affect the final shoulder balance of patients with EOS still needs to be further explored.

This study has several limitations. First, the aetiological diversity of EOS made it difficult to assess the efficacy considering the substantial variation in treatment indications and methods among different individuals. The patients included in this study were not only idiopathic but had a variety of curve types. Furthermore, there were some confounding factors leading to bias in this study, including different types of growing rods (single/dual rod(s)) and the replacement of a single rod by dual rods during the GR lengthening process. Moreover, the average follow-up period was relatively short, and more patients in this study were treated with a single rod rather than the most commonly used dual rod, which limited the significance of this study. A prospective multicentre study that focuses on a particular aetiology or type of curvature with more extended follow-up periods and a larger sample size should be developed to overcome the above limitations. Additionally, with the emergence of cosmetic shoulder balance research, it is worthwhile to study patient self-perception, self-evaluations of treatment efficacy, and satisfaction in addition to parameter accuracy and surgical outcomes.

Despite its shortcomings, this study still contributed several novel insights. This study was the first to observe and analyse the shoulder-balancing effect in a relatively large number of GR graduates who had completed final fusion, indicating that final fusion provided the opportunity to readjust the prefusion shoulder imbalance. Furthermore, the current results provide a wake-up call for spine surgeons to focus on the prefusion shoulder balance of GR graduates and to consider the importance of achieving shoulder balance in patients early after fusion. In practice, shoulder balance may not garner as much attention as spinal deformity correction or complication intervention. Extensive literature and a consensus on the surgical strategy for GR implantation and final fusion are still lacking. However, under the current conditions, the treatment results were satisfactory.

## Conclusions

Shoulder balance could be improved by GR implantation but often changes during the multiple lengthening processes, and the final result is relatively unpredictable. Final fusion could further adjust the prefusion shoulder imbalance. Focusing on the prefusion shoulder balance of GR graduates and providing patients with early shoulder balance after fusion might be necessary.

## Supplementary Information

**Additional file 1.**

## Data Availability

The datasets used and/or analysed during the current study are available from the corresponding author on reasonable request.

## Abbreviations

GR: Growing-rod; growing rod(s)
Imp: Implantation
RSH: Radiographic shoulder height
RSB: Radiographic shoulder balance
T1T: T1 tilt angle
CA: Clavicle angle
UIV: Upper instrumented vertebrae
LIV: Lower instrumented vertebrae
C7PL: Cervical 7 plumb line
CSVL: Centre sacral vertical line
SVA: Sagittal vertebral axis
PTC: Proximal thoracic curve
MC: Major curve
TK: Thoracic kyphosis
LL: Lumbar lordosis
