# Supplementary material for: Evaluation of changes in shoulder balance and prediction of final shoulder imbalance during growing-rod treatment for early-onset scoliosis
Source: BMC Musculoskelet Disord. 2021 Apr 14;22:354. doi: 10.1186/s12891-021-04221-9 (PMC8045187; doi:10.1186/s12891-021-04221-9)
Supplement: Supplementary file 1 — Additional file 1. [file 12891_2021_4221_MOESM1_ESM.docx]

**Evaluation of Changes in Shoulder Balance and Prediction of Final Shoulder Imbalance during Growing-Rod Treatment for Early-Onset Scoliosis**

Ziyang Liu, MD*^,1^; Tie Liu, MD*^,1^; Yong Hai, MD^1^; Lingyun Wu, MS^2^; Jonathan Junrui Hai^3^; Kang Gao, MS^1^; Xuanrong Guo, PhD^4^; Honghao Yang, MS^1^; Nan Kang, MD^1^; Fan Zhao, MD^1^

^1^Department of Orthopedic Surgery, Beijing Chaoyang Hospital, Capital Medical University, Beijing, China

^2^Karolinska Institutet, Stockholm, Sweden

^3^The High School Affiliated to Renmin University of China, Beijing, China

^4^University of Wisconsin Madison, Wisconsin, USA

*These authors contributed equally to this work

**Corresponding Author**

Yong Hai, MD

Department of Orthopedic Surgery, Beijing Chaoyang Hospital, Capital Medical University, Gongti North Rd, No. 8, Beijing 100020, China

Tel & Fax: +86-10-85231229

E-mail: prof.haiyong@yahoo.com; spinesurgeon@163.com

**Supplementary Material 1. Shoulder Balance Parental Perception and Parental Satisfaction Questionnaire: Developed for ‘Growing-Rod Graduates’**

**Name_________ Sex____ Date______ no. Patient_______**

**Please answer these questions according to the patient's situation**

1. **Before the first operation**, did you think your child had a situation in which the shoulders were not equal?

Please explain the extent

A. Severe shoulder imbalance

B. Mild shoulder imbalance

C. Basically normal shoulder contour

2. **Now**, do you think your child has a situation in which the shoulders are not equal?

Please explain the extent

A. Severe shoulder imbalance

B. Mild shoulder imbalance

C. Basically normal shoulder contour

3. Was there a change in shoulder balance **through the entire treatment period**?

Please explain the extent

A. Continued to improve

B. The balance had fluctuated, but it was generally better

C. No change

D. The balance had fluctuated, but it was generally worse

E. Continued to worsen

4. **Now**, are you **satisfied** with the patient's shoulder appearance?

Please explain the extent

A. Very satisfied

B. Basically satisfied

C. Not satisfied

D. Very dissatisfied

**Supplementary Material 2. Flowchart of the Procedures Adopted in this Study**
